# Fluoride therapy in the prevention of dental caries

**DOI:** 10.1186/1824-7288-40-S2-A19

**Published:** 2014-10-09

**Authors:** Laura Strohmenger

**Affiliations:** 1 Department Health’s Science, H.San Paolo, Milan, Italy

## 

The document "National Guidelines for the prevention and oral health promotion in childhood", 2013, is an act of address for those involved in the management of oral health and in particular to the pediatrician, because the figure of the pediatrician in the prevention of oral health is absolutely critical, as confirmed by the entire international literature in recent years.

Correct attitudes and behaviors adopted since childhood that will allow the child to protect his health.

The fluoride is the cornerstone of prevention of tooth decay and is required for all individuals.

Over the years, have been developed different means of administration of fluorine, each with different strengths, dosages and frequency of use.

Fluoride supplements should be prescribed by the pediatrician in cases of real difficulty for topical administration of fluoride through toothpaste or fluoride added as a method of in subjects at risk of tooth decay.

The decline of caries in our country it is highly likely also due to the pediatrician who, using the national guidelines, can inform parents and families induce the acquisition of preventive behaviors currently defined by scientific research.

To this end, again, the ministerial guidelines on the subject that were reviewed by a team of experts representative of the Italian research in this area.
